# Characterization of *Yersinia enterocolitica* Biotype 1A Strains Isolated from Swine Slaughterhouses and Markets

**DOI:** 10.1155/2013/769097

**Published:** 2013-02-17

**Authors:** Renata Paixão, Luisa Zanolli Moreno, Débora Dirani Sena de Gobbi, Daniele Cristine Raimundo, Ernesto Hofer, Maria Helena Matté, Thais Sebastiana Porfida Ferreira, Vasco Tulio de Moura Gomes, Barbara Leticia Pereira Costa, Andrea Micke Moreno

**Affiliations:** ^1^Departamento de Medicina Veterinária Preventiva e Saúde Animal, Faculdade de Medicina Veterinária e Zootecnia, Universidade de São Paulo, Avenida Prof. Dr. Orlando Marques de Paiva 87, Butanta, 05508-270 São Paulo, SP, Brazil; ^2^Faculdade de Medicina Veterinária, Faculdades Metropolitanas Unidas (FMU), Rua Ministro Nelson Hungria 541, 05690-050 São Paulo, SP, Brazil; ^3^Laboratório de Saúde Pública, Faculdade de Saúde Pública, Universidade de São Paulo, Avenida Dr. Arnaldo 715, 01246-904 São Paulo, SP, Brazil; ^4^Laboratório de Zoonoses Bacterianas, Fundação Instituto Oswaldo Cruz (FIOCRUZ), Avenida Brasil 4365, 21045-900 Rio de Janeiro, RJ, Brazil

## Abstract

*Yersinia enterocolitica* is an important foodborne pathogen that causes illness in humans and animals. *Y. enterocolitica* is also the most heterogeneous species of the genus and is divided into distinct serotypes and over six biotypes. *Y. enterocolitica* biotype 1A strains are classically considered as nonpathogenic; however, some biotype 1A isolates have been considered as causative of gastrointestinal disease, yielding symptoms indistinguishable from those produced by pathogenic biotypes. Even after decades of isolation of clinical strains, the pathogenic mechanisms of these isolates are still not fully understood. In the present study, 122 *Yersinia enterocolitica* biotype 1A strains isolated from swine slaughterhouses and meat markets in Sao Paulo, Brazil, were characterized according to the presence of the virulence genes *ail*, *vir*F, and *yst*A. A total of 94 strains were positive to at least one virulence gene (77.05%), and 67 were positive to all of them (54.92%). Twenty-two strains were submitted to PFGE genotyping resulting in 22 distinct pulsotypes, varying from 50% to 84% of genetic similarity. Any clustering tendency among pulsotypes related to origin, isolation site, or even virulence profile was not observed. The present study reports an important contamination of the environment in swine slaughterhouses, meat markets, and pork, by potentially virulent *Y. enterocolitica* biotype 1A.

## 1. Introduction

The *Yersinia* genus belongs to the *Enterobacteriaceae* family, and among its 15 species, *Yersinia enterocolitica* is the most prevalent cause of illness in humans and animals [1, 2]. It is an important foodborne pathogen, causing acute diarrhea, terminal ileitis, mesenteric lymphadenitis, and long-term sequelae that may follow the infection [1, 3]. *Y. enterocolitica* is also the most heterogeneous species of the genus, and it is divided into distinct serotypes and six biotypes [4]. Most of the pathogenic lineages belong to biotypes 1B, 2, 3, 4, and 5, while environmental strains considered nonpathogenic to humans and animals belong to biotype 1A [3, 5].

The virulence of pathogenic biotypes is attributed to the presence of plasmidial and chromosomal genes. The virulence plasmid of *Yersinia *(pYV) encodes adhesin A (*Yad*A), *Yersinia* outer proteins (*Yops*) from the type III secretion system, and transcriptional regulator gene (*vir*F) [6, 7]. The chromosomal virulence genes include invasin (*inv*), attachment and invasion locus (*ail*), *Yersinia* stable toxin A (*yst*A), and mucoid *Yersinia* factor A (*myf*A) [8]. Some of these factors are restricted to pathogenic pYV-bearing strains of *Y. enterocolitica*, such as *ail*, *yst*A, and *myf*A, while the *inv* gene is common to pathogenic and nonpathogenic strains [5].

*Y. enterocolitica* biotype 1A strains are classically considered as nonpathogenic, since they do not bear pYV plasmid and chromosomal virulence genes, such as *ail*, *myf*A, *yst*A, and the *ysa* locus [9–11]. However, some biotype 1A strains have been considered as causative of gastrointestinal disease, yielding symptoms indistinguishable from those produced by pathogenic biotypes [12, 13]. Infection by biotype 1A strains may persist for several weeks or months, and it is frequent for all age groups, in contrast to pYV-bearing strains, which are mostly recurrent in children [14, 15].

Biotype 1A *yersiniae* have been associated with nosocomial [16] and foodborne [17] outbreaks of gastrointestinal infection. It was also isolated from several animal species used in human nourishment [18, 19]. Even after decades of clinical strains isolation, the pathogenic mechanisms of these strains remain not fully understood. It has been detected that some clinical strains of biotype 1A bear homologous sequences to the *ail*, *myf*A, and *yst*A genes, which were considered to be restricted to pathogenic biotypes [5, 20–23]. 

Distinct techniques of genotyping presented a tendency to cluster biotype 1A strains, isolated from various sources into two clonal groups; both of them were represented by clinical and nonclinical isolates [24]. Falcão et al. [5] described the first biotype 1A food isolate in Brazil that bore the *ail* and *yst*A genes, and it was grouped closer to strains of human and animal clinical material by pulsed-field gel electrophoresis (PFGE) technique. The aim of this study was to characterize *Yersinia enterocolitica* biotype 1A isolated from swine slaughterhouses and markets in São Paulo, Brazil.

## 2. Material and Methods

### 2.1. Culture Collection Strains

The following strains were used as positive and negative controls for biochemical and PCR tests: *Yersinia enterocolitica* O:3 biotype 4 (MyO—SW/897/63), *Y. enterocolitica* O:8 biotype 1B (P311—WF—Albany, USA), *Y. enterocolitica* O:9 biotype 2 (My79—Nilhén, Sweden), *Y. pseudotuberculosis*—IAL1791, *Y. frederiksenii*—CIP8029, and *Y. kristensenii*—CIP9993, all of them from the Laboratory of Bacterial Zoonoses, Bacteriology Department of Oswaldo Cruz Institute, RJ, Brazil (IOC/FIOCRUZ).

### 2.2. Sampling and Microbiological Analysis

A total of 12 collects were carried out between 2007 and 2008 in two swine slaughterhouses and two respective markets in São Paulo State, Brazil. A total of 792 samples were collected, including 480 swabs from tonsils and tongue, 120 swabs from slaughterhouse environment points, 72 swabs from market environment points, and 120 pork fragments. Tonsils and tongue and environment swabs were performed using sterile sponges (Whirl-Pak Speci-Sponge bag—NASCO, EUA—11.5 cm × 23 cm), hydrated with 20 ml of Letheen Broth (Difco/BBL, Detroit, MI, USA). From each environmental site (wall, table, or floor), a 100 cm^2^ area was also collected. Samples were kept under refrigeration until laboratory processing.

The samples were processed with cold enrichment with phosphate-buffered saline, sorbitol, and bile salts number 3 (Difco/BBL, Detroit, MI, USA) for 10 to 12 days. An aliquot of the broth (10 *μ*L) was treated with potassium hydroxide (KOH), and then a loopful was plated onto MacConkey (Difco/BBL, Detroit, MI, USA) and cefsulodin-irgasan-novobiocin (CIN) agar (Difco/BBL, Detroit, MI, USA). Plates were incubated for 24 h at 30°C, under aerobic conditions. At least five colonies presenting suggestive morphology were selected from each selective agar for biochemical identification, including the Kligler iron and Christensen urea tests, fermentation of sucrose, rhamnose, and melibiose.

Strains positive to biochemical identification were biotyped according to the reduced biotyping schema proposed by Souza et al. [2]. Strains classified as *Y. enterocolitica* biotype 1A were submitted to the virulence gene detection through PCR and genotyping by PFGE.

### 2.3. DNA Preparation and Virulence Genes Detection

An aliquot of 1 mL of *Yersinia enterocolitica* fresh culture in brain heart infusion—BHI (Difco/BBL, Detroit, MI, USA)—was harvested by centrifugation at 4,000 ×g for 5 min. The pellet was submitted to DNA extraction, based on the method described by Boom et al. [25]. The DNA samples were amplified by simultaneous detection of the *ail, vir*F, and *yst*A genes, as described by Lambertz and Danielsson-Tham [26]. Amplification was carried out in a 50 *μ*L reaction mixture, containing 5 *μ*L of DNA template, 1.5 mM of MgCl_2_, 200 mM of each dNTP, 20 *ρ*mol of each primer and 1 U of Taq DNA polymerase, 1X PCR buffer, and ultrapure water. 

Amplification conditions were as follows: an initial denaturation at 94°C for 3 min, followed by 30 cycles of denaturation at 94°C for 30 s, annealing at 60°C for 1 min, and extension at 72°C for 1 min, with a final extension at 72°C for 5 min. PCR products were separated in 2% agarose gel stained with BlueGreen (LGC Biotecnologia, São Paulo, Brazil) and identified using 100 bp DNA Ladder. 

### 2.4. PFGE Typing

Twenty-two pure colonies of *Y. enterocolitica* 1A with different origins and virulence profiles were submitted for PFGE genotyping. DNA was extracted from 6 mL of overnight culture as previously described [5].

The DNA was digested for 4 h with 6 U *Not*I (New England BioLabs Inc., Ipswich, MA, USA), and the restriction fragments were separated on a 1.0% pulsed-field-certified agarose (Bio-Rad Laboratories, CA, USA) in 0.5X Tris-borate EDTA (TBE) using a CHEF-DRIII system (Bio-Rad Laboratories). Pulse times were ramped from 1 to 18 s over 20 h, using an electric field of 6 V/cm, at a 120° angle at 14°C. The gels were stained with SYBR Safe (Invitrogen Corporation, CA, USA) for 40 min and photographed under UV transillumination. DNA fragments were identified using Lambda DNA-PFGE marker (New England BioLabs Inc., USA).

### 2.5. Statistical Analysis

The levels of relatedness of the strains were determined by comprehensive pairwise comparison of restriction fragment sizes, using Dice coefficient. Mean values obtained from Dice coefficients were employed in UPGMA, using BioNumeric 6.6 (Applied Maths) to generate dendrograms. For PFGE analysis, strains were considered as part of different subtypes, when differing by four or more bands. 

## 3. Results

From the 792 samples collected, 442 *Y. enterocolitica* strains were recovered and bioserotyped. Out of these, 92 were identified as *Y. enterocolitica* 1A/nontypeable (20.81%), 10 *Y. enterocolitica* 1A/O:5a (2.26%), 18 *Y. enterocolitica* 1A/O:5b (4.07%), one *Y. enterocolitica *1A/O:7 (0.23%), and one *Y. enterocolitica* 1A/O:6 (0.23%). The other 320 (72.40%) strains were identified as bioserotype 4/O:3. All 122 biotype 1A strains were isolated from pork, markets, or slaughterhouses environments (Table 1). None of the tonsils and tongue swabs were positive to *Y. enterocolitica* 1A isolation; only *Y. enterocolitica *4/O:3 was found in these samples (data not shown).

A higher occurrence of *Y. enterocolitica* biotype 1A in the environment of production line 2 (slaughterhouse and market 2) was observed, with predominance of nontypeable strains (Table 1). Pork originated from this production line also presented a higher contamination by *Y. enterocolitica* biotype 1A, especially in relation to nontypeable strains (78.57%; 22/28). Production line 1 presented a lower contamination by biotype 1A; market 1 presented the lowest recovery of *yersiniae* with isolation of only 9 strains of *Y. enterocolitica* biotype 1A.

Research on virulence genes revealed, at electrophoresis, that positive strains presented a 454 bp band to the *ail* gene, 700 bp to *vir*F, and 145 bp to the *yst*A gene. From the 122 strains of *Y. enterocolitica* biotype 1A, 77.05% were positive to at least one virulence gene, and 54.92% were positive to all of them (Table 2). Most of nontypeable strains were positive to all three virulence genes or just presented the *vir*F gene. *Y. enterocolitica* 1A serotypes O:5a and O:5b presented a lower variation of virulence genes than nontypeable strains, with a high frequency of positivity to the *vir*F*, ail,* and *yst*A genes. The serotypes O:6 and O:7 were negative to all genes analysed. 

**Figure 1:**
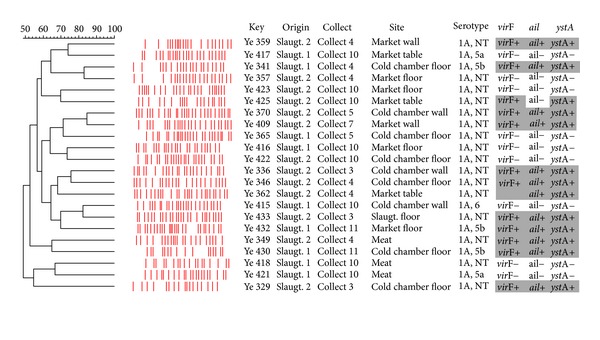
Dendrogram showing comparison of *Y. enterocolitica* biotype 1A strains through PFGE.

PFGE genotyping resulted in 22 distinct pulsotypes varying from 50% to 84% of genetic similarity. Pulsotypes presented a greater genetic heterogeneity, as demonstrated by the dendrogram in Figure 1. Any clustering tendency among pulsotypes related to origin, isolation site, or even virulence profile was not observed. Persistent pulsotypes in sequential collects and samples were not detected.

## 4. Discussion

From the six biotypes of *Y. enterocolitica*, biotype 1A is the most heterogeneous, and its most common serotypes are O:5, O:6,30, O:6,31, O:7,8, and O:10, as well as nontypeable strains [11]. It is a ubiquitous biotype that has been isolated from distinct types of environment, such as soil and various sources of water and food, including vegetables and animal products, and it was also isolated from different animal species [18, 27–29]. In this study, *Y. enterocolitica* biotype 1A was isolated from different environmental sites (wall, table, and floor) of swine slaughterhouses and markets and also from pork. Contamination of animal (tonsils and tongue) was detected in only one of the slaughterhouses studied and in a low percentage. 

Serotype O:5 and nontypeable strains were the most prevalent among the samples analyzed, which is compliant with the literature [11]. On punctual collects, serotypes O:6 and O:7 strains were also detected. Persistency of the serotypes over the sequential collects, or their continuity among production lines was not observed. These facts, as well as the low frequency of animal contamination, suggest that the source of environmental contamination is probably external to the production line, such as employees or even water and other fomites. The ubiquitous nature of this biotype allows it to be carried to the food processing industry environment, thus contaminating the food intended for consumption and representing a risk to consumers health.

The genetic heterogeneity of pulsotypes also confirm the possibility of external contamination sources to the environment of the slaughterhouses and markets, since persistency of specific pulsotypes over the production lines or sequential collects was not detected. The diversity of biotype 1A PFGE profiles was expected and corroborates the literature that classifies this biotype as the most heterogeneous, with strains of the same serotype presenting considerable genetic diversity, whereas pathogenic pYV-bearing bioserotypes are usually considered relatively stable [11, 30, 31].

The characteristic avirulence of *Y. enterocolitica* biotype 1A strains is traditionally conditioned to the absence of important virulence genes, as well as to the high prevalence of this biotype strains in the environment and in healthy animals [11]. Nevertheless, the isolation of this biotype among clinical samples from diarrheic patients still intrigues many researchers as to the pathogenic potential of these strains. In the present study, plasmidial and chromosomal virulence genes were detected in environmental and pork strains of *Y. enterocolitica* biotype 1A.

From the virulence genes studied, plasmidial gene *vir*F is considered rarely present in *Y. enterocolitica* biotype 1A strains [22]; it was detected, however, in 71.31% (87/122) of the analyzed strains. Zheng et al. [32] also reported a high frequency of the *vir*F gene among biotype 1A *yersiniae*, which contradicts the findings of Bhagat and Virdi [22] and the typical classification of nonpathogenic biotype 1A that lacks plasmidial genes [9–11]. 

The chromosomal genes *ail* and *yst*A were also found with high frequency in the studied strains. The *yst* genes, which originate heat-stable enterotoxins, such as Yst-a and Yst-b, have been previously described in *Y. enterocolitica* biotype 1A strains [5, 32, 33], although it has been suggested that these genes may be nonfunctional in some biotype 1A strains [11]. Nevertheless, Singh and Virdi [33] reported that Yst-b can be produced in the ileum environment, thus suggesting that it can be an important virulence factor for *Y. enterocolitica* biotype 1A strains. 

The *ail* gene has been described as a stable virulence marker that has a high correlation with virulent *Y. enterocolitica *[34]. For this reason, detection methods based on the *ail* gene have been developed [35, 36]. However, there have been previous reports of sporadic biotype 1A strains positive to *ail*-specific PCR, such as the present study [5, 21, 23, 37]. Therefore, if the use the of *ail* gene alone as a detection method for pathogenic *Y. enterocolitica *continues, there is risk of misidentification of pathogenic bioserotypes and the continuity of subnotification of virulent biotype 1A strains [23]. 

The present study reported an important contamination of the environment of swine slaughterhouses and markets by *Y. enterocolitica* biotype 1A. This contamination was not introduced by animal, as reported previously; it is probably due to external contamination from environment or carried by the employees. Even though the continuity of serotypes or pulsotypes over the production lines was not detected, the magnitude of market and pork contamination represents a risk to the consumers' health. This risk is confirmed and amplified by the high frequency of positive strains to the virulence genes *vir*F*, ail,* and *yst*A, which can present pathogenic potential to humans. 

## Figures and Tables

**Table 1:** Distribution of *Yersinia enterocolitica *1A  biotype according to sample origin and isolation site.

	Slaughterhouse 1		Slaughterhouse 2		Market 1		Market 2	
	Animal*	Envir.**	Animal	Envir.	Pork	Envir.	Pork	Envir.
1A/NT	0	1	2	18	4	2	22	43
1A/O:5a	0	1	0	2	1	1	2	3
1A/O:5b	0	6	0	2	0	1	4	5
1A/O:6	0	1	0	0	0	0	0	0
1A/O:7	0	0	0	0	0	0	0	1

Total	0	9	2	22	5	4	28	52

*Tonsils and tongue swab; **environment.

**Table 2:** Virulence gene profiles generated by multiplex PCR in *Yersinia enterocolitica *1A biotype.

	Virulence gene profile		1A/NT	1A/O:5a	1A/O:5b	1A/O:7	1A/O:6	Total
*vir*F +	*ail* +	*yst*A +	44	7	16	—	—	67
*vir*F −	*ail* +	*yst*A +	3	—	1	—	—	4
*vir*F +	*ail* −	*yst*A +	1	—	—	—	—	1
*vir*F +	*ail* +	*yst*A −	1	—	1	—	—	2
*vir*F +	*ail* −	*yst*A −	17	—	—	—	—	17
*vir*F −	*ail* −	*yst*A +	1	—	—	—	—	1
*vir*F −	*ail* +	*yst*A −	2	—	—	—	—	2
*vir*F −	*ail* −	*yst*A −	23	3	—	1	1	28

Total			92	10	18	1	1	122

## References

[1] Bottone EJ (1999). *Yersinia enterocolitica*: overview and epidemiologic correlates. *Microbes and Infection*.

[2] Souza RA, Falcão DP, Falcão JP (2011). Emended description of *Yersinia* massiliensis. *International Journal of Systematic and Evolutionary Microbiology*.

[3] Robins RM, Doyle MP, Beuchat LR, Montiville TJ (2001). Yersinia enterocolitica. *Food Microbiology: Fundamentals and Frontiers*.

[4] Wauters G, Kandolo K, Janssens M (1987). Revised biogrouping scheme of *Yersinia enterocolitica*. *Contributions to Microbiology and Immunology*.

[5] Falcão JP, Falcão DP, Pitondo-Silva A, Malaspina AC, Brocchi M (2006). Molecular typing and virulence markers of *Yersinia enterocolitica* strains from human, animal and food origins isolated between 1968 and 2000 in Brazil. *Journal of Medical Microbiology*.

[6] Cornelis GR, Boland A, Boyd AP (1998). The virulence plasmid of *Yersinia*, an antihost genome. *Microbiology and Molecular Biology Reviews*.

[7] Cornelis GR (2001). The *Yersinia* YSC-Yop “type III” weaponry. *Nature Reviews Molecular Cell Biology*.

[8] Revell PA, Miller VL (2001). *Yersinia* virulence: more than a plasmid. *FEMS Microbiology Letters*.

[9] Robins-Browne RM, Miliotis MD, Cianciosi S, Miller VL, Falkow S, Morris JG (1989). Evaluation of DNA colony hybridization and other techniques for detection of virulence in *Yersinia *species. *Journal of Clinical Microbiology*.

[10] Foultier B, Troisfontaines P, Müller S, Opperdoes FR, Cornelis GR (2002). Characterization of the ysa pathogenicity locus in the chromosome of *Yersinia enterocolitica* and phylogeny analysis of type III secretion systems. *Journal of Molecular Evolution*.

[11] Tennant SM, Grant TH, Robins-Browne RM (2003). Pathogenicity of *Yersinia enterocolitica* biotype 1A. *FEMS Immunology and Medical Microbiology*.

[12] Morris JG, Prado V, Ferreccio C (1991). *Yersinia enterocolitica* isolated from two cohorts of young children in Santiago, Chile: incidence of and lack of correlation between illness and proposed virulence factors. *Journal of Clinical Microbiology*.

[13] Burnens AP, Frey A, Nicolet J (1996). Association between clinical presentation, biogroups and virulence attributes of *Yersinia enterocolitica* strains in human diarrhoeal disease. *Epidemiology and Infection*.

[14] Stolk-Engelaar VMM, Hoogkamp-Korstanje JAA (1996). Clinical presentation and diagnosis of gastrointestinal infections by *Yersinia enterocolitica* in 261 Dutch patients. *Scandinavian Journal of Infectious Diseases*.

[15] Lobato MJ, Landeras E, González-Hevia MA, Mendoza MC (1998). Genetic heterogeneity of clinical strains of *Yersinia enterocolitica* traced by ribotyping and relationships between ribotypes, serotypes, and biotypes. *Journal of Clinical Microbiology*.

[16] Ratnam S, Mercer E, Picco B (1982). A nosocomial outbreak of diarrheal disease due to *Yersinia enterocolitica* serotype O:5, biotype 1. *Journal of Infectious Diseases*.

[17] Greenwood MH, Hooper WL (1990). Excretion of *Yersinia* spp. associated with consumption of pasteurized milk. *Epidemiology and Infection*.

[18] McNally A, Cheasty T, Fearnley C (2004). Comparison of the biotypes of *Yersinia enterocolitica* isolated from pigs, cattle and sheep at slaughter and from humans with yersiniosis in Great Britain during 1999-2000. *Letters in Applied Microbiology*.

[19] Arnold T, Neubauer H, Ganter M (2006). Prevalence of *Yersinia enterocolitica* in goat herds from northern Germany. *Journal of Veterinary Medicine B*.

[20] Grant T, Bennett-Wood V, Robins-Browne RM (1998). Identification of virulence-associated characteristics in clinical isolates of *Yersinia enterocolitica* lacking classical virulence markers. *Infection and Immunity*.

[21] Thoerner P, Kingombe CIB, Bögli-Stuber K (2003). PCR detection of virulence genes in *Yersinia enterocolitica* and *Yersinia pseudotuberculosis* and investigation of virulence gene distribution. *Applied and Environmental Microbiology*.

[22] Bhagat N, Virdi JS (2007). Distribution of virulence-associated genes in *Yersinia enterocolitica* biovar 1A correlates with clonal groups and not the source of isolation. *FEMS Microbiology Letters*.

[23] Sihvonen LM, Toivonen S, Haukka K, Kuusi M, Skurnik M, Siitonen A (2011). Multilocus variable-number tandem-repeat analysis, pulsed-field gel electrophoresis, and antimicrobial susceptibility patterns in discrimination of sporadic and outbreak-related strains of *Yersinia enterocolitica*. *BMC Microbiology*.

[24] Sachdeva P, Virdi JS (2004). Repetitive elements sequence (REP/ERIC)-PCR based genotyping of clinical and environmental strains of *Yersinia enterocolitica* biotype 1A reveal existence of limited number of clonal groups. *FEMS Microbiology Letters*.

[25] Boom R, Sol CJA, Salimans MMM (1990). Rapid and simple method for purification of nucleic acids. *Journal of Clinical Microbiology*.

[26] Lambertz ST, Danielsson-Tham ML (2005). Identification and characterization of pathogenic *Yersinia enterocolitica* isolates by PCR and pulsed-field gel electrophoresis. *Applied and Environmental Microbiology*.

[27] Harvey S, Greenwood JR, Pickett MJ, Mah RA (1976). Recovery of *Yersinia enterocolitica* from streams and lakes of California. *Applied and Environmental Microbiology*.

[28] Shayegani M, DeForge I, McGlynn DM, Root T (1981). Characteristics of Yersinia enterocolitica and related species isolated from human, animal, and environmental sources. *Journal of Clinical Microbiology*.

[29] Sulakvelidze A, Dalakishvili K, Barry E (1996). Analysis of clinical and environmental *Yersinia* isolates in the Republic of Georgia. *Journal of Clinical Microbiology*.

[30] Najdenski H, Iteman I, Carniel E (1994). Efficient subtyping of pathogenic *Yersinia enterocolitica* strains by pulsed-field gel electrophoresis. *Journal of Clinical Microbiology*.

[31] Iteman I, Guiyoule A, Carniel E (1996). Comparison of three molecular methods for typing and subtyping pathogenic *Yersinia enterocolitica* strains. *Journal of Medical Microbiology*.

[32] Zheng H, Sun Y, Mao Z, Jiang B (2008). Investigation of virulence genes in clinical isolates of *Yersinia enterocolitica*. *FEMS Immunology and Medical Microbiology*.

[33] Singh I, Virdi JS (2004). Production of *Yersinia* stable toxin (YST) and distribution of yst genes in biotype 1A strains of *Yersinia enterocolitica*. *Journal of Medical Microbiology*.

[34] Miller VL, Farmer JJ, Hill WE, Falkow S (1989). The aid locus is found uniquely in *Yersinia enterocolitica* serotypes commonly associated with disease. *Infection and Immunity*.

[35] Wannet JBW, Reessink M, Brunings HA (2001). Detection of pathogenic *Yersinia enterocolitica* by a rapid and sensitive Duplex PCR assay. *Journal of Clinical Microbiology*.

[36] Lambertz ST, Nilsson C, Hallanvuo S, Lindblad M (2008). Real-time PCR method for detection of pathogenic *Yersinia enterocolitica* in food. *Applied and Environmental Microbiology*.

[37] Cheyne BM, Van Dyke MI, Anderson WB, Huck PM (2010). The detection of *Yersinia enterocolitica* in surface water by quantitative PCR amplification of the ail and yadA genes. *Journal of Water and Health*.

